# The family Ctenobelbidae (Acari, Oribatida), with description of a new species and discussion on systematic placement and taxonomic status of the genus *Berndamerus* Mahunka, 1977

**DOI:** 10.3897/zookeys.395.7224

**Published:** 2014-04-01

**Authors:** Sergey G. Ermilov, Umukusum Ya. Shtanchaeva, Luis S. Subías, Alexander E. Anichkin

**Affiliations:** 1 Tyumen State University, Tyumen, Russia; 2 Joint Russian-Vietnamese Tropical Research and Technological Center, Hanoi-Ho Chi Minh, Vietnam; 3 Complutense University, Madrid, Spain; 4 A.N. Severtsov Institute of Problems of Ecology and Evolution, Russian Academy of Sciences, Moscow, Russia

**Keywords:** Oribatid mites, Ctenobelbidae, *Ctenobelba*, *Berndamerus*, new status, new combination, new species, key, Vietnam

## Abstract

The oribatid mite genus *Berndamerus* Mahunka, 1977 is transferred into the family Ctenobelbidae as the subgenus *Ctenobelba (Berndamerus)* Mahunka, 1977, **stat. n.** from the family Amerobelbidae. The known species of *Berndamerus* combined: *C. (B.) bicostata* (Berlese, 1910), **comb. n.**, *C. (B.) eremuloides* (Berlese, 1910), **comb. n.**, *C. (B.) hellenica* (Mahunka, 1977), **comb. n.** A new species, *Ctenobelba (Berndamerus) bugiamapensis* **sp. n.**, is described from soil, Bu Gia Map National Park, southern Vietnam. It differs from the other species of the subgenus by the heterotrichy of notogastral setae, presence of adanal neotrichy and localization of adanal lyrifissures. Ctenobelbidae is recorded in Vietnam for the first time. A new diagnosis of the family Ctenobelbidae and the identification keys to the known subgenera of the genus *Ctenobelba* and species of the subgenus *Ctenobelba (Berndamerus)* are provided.

## Introduction

The family Ctenobelbidae is monotypic, presented by the genus *Ctenobelba* Balogh, 1943 (Balogh 1943) with *Eremobelba pectinigera* Berlese, 1908 (Berlese 1908) as type species. Currently, the genus *Ctenobelba* comprises 32 species, which are distributed in the Palaearctic and Oriental regions (Subías 2004, online version 2013), and five subgenera: *Ctenobelba (Ctenobelba)* Balogh, 1943 (with 22 species), *Ctenobelba (Aokibelba)* Subías & Shtanchaeva, 2013 (five species), *Ctenobelba (Berndamerus)* Mahunka, 1977, stat. n. (three species), *Ctenobelba (Bifurcobelba)* Subías & Shtanchaeva, 2010 (one species) and *Ctenobelba (Caucasiobelba)* Subías & Shtanchaeva, 2010 (one species). The main morphological characters of *Ctenobelba (Berndamerus)* stat. n. were summarized by Mahunka (1977); *Ctenobelba (Aokibelba)*, *Ctenobelba (Bifurcobelba)* and *Ctenobelba (Caucasiobelba)* – by Subías and Shtanchaeva (2010, 2013).

The primary purpose of paper is to discuss the systematic placement and taxonomic status of the subgenus *Ctenobelba (Berndamerus)* stat. n., which was described as the genus *Berndamerus* Mahunka, 1977 of the family Amerobelbidae.

During taxonomic survey of oribatid fauna of the Bu Gia Map National Park in southern Vietnam (a brief geographical and floristic descriptions of this park was given earlier – Ermilov et al. 2012a), we found a new species of *Ctenobelba (Berndamerus)* stat. n. It is a first representative of the Ctenobelbidae recorded from Vietnam. The secondary purpose of the present paper is to describe and illustrate this new species.

We provide a new diagnosis of the family Ctenobelbidae and present the identification keys to the known subgenera of the genus *Ctenobelba* and species of the subgenus *Ctenobelba (Berndamerus)* stat. n.

## Material and methods

Six specimens (holotype: male; five paratypes: four females and one male) of *Ctenobelba (Berndamerus) bugiamapensis* sp. n.: southern Vietnam, Binh Phuoc Province, Bu Gia Map National Park, 12°12'N, 107°12'E, 350 m a.s.l., dark loamy soil, 13.XI.2013 (collected by A.E. Anichkin and S.G. Ermilov).

Soil samples were collected by taking 10 soil cores (diameter: 7.8 cm; depth: 10 cm). The samples collected were left in the metal cores to minimize disturbance during transportation from the field to the laboratory. Oribatid mites were extracted into 75 per cent ethanol using Berlese’s funnels with electric lamps (40 W) during ten days. Specimens of the new species were found in three samples out of 10.

Holotype and paratypes were mounted in lactic acid on temporary cavity slides for measurement and illustration. The body length was measured in lateral view, from the tip of the rostrum to the posterior edge of the ventral plate. The notogastral width refers to the maximum width in dorsal aspect (without pteromorphs). Lengths of body setae were measured in lateral aspect. All body measurements are presented in micrometers. Formulae for leg setation are given in parentheses according to the sequence trochanter–femur–genu–tibia–tarsus (famulus included). Formulae for leg solenidia are given in square brackets according to the sequence genu–tibia–tarsus. General terminology used in this paper follows that of Grandjean (summarized by Norton and Behan-Pelletier 2009).

## Results

### Systematic placement and taxonomic status of the genus *Berndamerus* Mahunka, 1977

Mahunka (1977) described the genus *Berndamerus* with *Berndamerus hellenicus* Mahunka, 1977, as the type species, and included it in the family Amerobelbidae. Later, Mahunka and Mahunka-Papp (1995), Subías (2004), Ermilov (2011) supported *Berndamerus* in this family.

However, mites of the family Amerobelbidae differ from those of the family Ctenobelbidae by an important morphological character – absence (versus presence) of prodorsal costulae (Norton and Behan-Pelletier 2009). All species of *Berndamerus* are with well developed costulae. Therefore inclusion of this genus in Amerobelbidae is doubtful. Thus, we suggest the genus *Berndamerus* should be transferred to the family Ctenobelbidae.

The main generic morphological character of *Berndamerus* is the arrangement of notogastral setae (located dorsally, usually in two longitudinal rows). This character state is not an apomorphy, because it is inherent also for some other taxa in the Ameroidea, including the ctenobelbid subgenus *Ctenobelba (Caucasiobelba)* Subías & Shtanchaeva, 2010. Thus, the generic status of *Berndamerus* cannot be supported, and we consider that it should be included as the subgenus in the genus *Ctenobelba*, differing from *Ctenobelba (Caucasiobelba)* by the absence of reticulate body surface: *Ctenobelba (Berndamerus)* Mahunka, 1977 stat. n. Hence, all species of *Berndamerus* also should be combined in *Ctenobelba (Berndamerus)*: *Ctenobelba (Berndamerus) bicostata* (Berlese, 1910) comb. n. (see Berlese 1910a; Mahunka and Mahunka-Papp 1995), *Ctenobelba (Berndamerus) eremuloides* (Berlese, 1910) comb. n. (see Berlese 1910b; Mahunka and Mahunka-Papp 1995), *Ctenobelba (Berndamerus) hellenica* (Mahunka, 1977) comb. n. (see Mahunka 1977).

#### Ctenobelba (Berndamerus) bugiamapensis sp. n.

http://zoobank.org/03D1D2DB-8CE5-441F-B7CA-E95A4C5D5980

http://species-id.net/wiki/Ctenobelba_bugiamapensis

Figs 1 – 9

##### Diagnosis.

Body length 315–365 × 199–232. Dorsal body surface smooth, ventral body surface microfoveolate. Rostrum rounded. Transcostula poorly developed. All prodorsal setae setiform, barbed; lamellar setae longest and thickest. Sensilli setiform, ciliate Notogastral setae *c* longest, barbed; *p*_1_–*p*_3_ of medium size, barbed; other setae short, smooth. Setae *c*, *la*, *lm* inserted in one longitudinal row; *lp*, *h*_2_, *h*_3_ inserted close to each other. Aggenito-adanal neotrichy present: eight pairs of setiform, barbed setae developed.

##### Description.

*Measurements*. Body length: 348 (holotype), 315–365 (five paratypes); notogaster width: 215 (holotype), 199–232 (five paratypes).

*Integument*. Body color yellow-brownish to brown. Dorsal body surface smooth; ventral body surface (including subcapitular mentum, genital and anal plates) microfoveolate (diameter of foveolae up to 0.5), which is visible only under high magnification (× 1500). The region adjacent to the anal aperture striate.

*Prodorsum*. Rostrum rounded. Costulae (*cos*) well developed, almost straight, as long as half of prodorsum (in lateral view). Transcostula (*tcos*) present, poorly visible. All prodorsal setae setiform, barbed: rostral setae (*ro*, 36–41) thin, inserted dorso-laterally; interlamellar setae (*in*, 73–82) slightly thicker than rostral setae, inserted posteriorly to costulae; lamellar setae longest (*le*, 94–98) and thickest, inserted on the costular tips; exobothridial setae shortest (*ex*, 24–32) and thinnest, inserted laterally on prodorsum. Sensilli (*ss*, 114–118) setiform, thickened, ciliate unilaterally. Lateral carinae (not tutoria) distinct (*car*).

*Notogaster*. Anterior border straight, slightly developed, with one pair of blunt-ended medial condyles. One pair of short humeral cristae present. Ten pairs of notogastral setae present: *c* (36–41) and *p*_1_–*p*_3_ (18–20) setiform, thin, barbed; other setae short (6–8), smooth. Setae *c*, *la*, *lm*, *lp*, *h*_2_, *h*_3_ located dorsally: *c*, *la*, *lm* inserted in one longitudinal row; *lp*, *h*_2_, *h*_3_ inserted close to each other. Setae *h*_1_ located dorso-posteriorly. Setae *p*_1_–*p*_3_ located posteriorly, in one transverse row; *p*_1_ inserted close to each other. Opisthonotal gland openings (*gla*) small, located laterally to setae *lm*. Lyrifissures *ia* located posteriorly to cristae. Lyrifissures *im*, *ip*, *ih* located dorso-laterally, *ips*–posteriorly; all nearly to muscle sigillar band. Circumventral carina (*cv*) distinct.

*Gnathosoma*. Morphology of subcapitulum, palps and chelicerae typical for *Ctenobelba* (Ermilov et al. 2012b). Subcapitulum longer than wide: 73–82 × 53–57. Subcapitular setae setiform, barbed; *h* and *m* (both 24–26) longer than *a* (12–16). Adoral setae absent. Palps (49–53) with setation 0–2–1–3–8(+ω). Solenidion long, thickened, not fused with *acm*, pressed to surface of palptarsus. Chelicerae (82–86) with two long, setiform, barbed setae; *cha* (24–26) longer than *chb* (16–18). Trägårdh’s organ (Tg) distinct.

*Lateropodosomal and epimeral regions*. Pedotecta I (Pd I), II (Pd II) large, scale-like. Apodemes I, II well developed; sejugal apodemes absent, represented only epimeral borders. Epimeral setal formula: 3–1–3–3. Setae setiform, barbed: *1b*, *1c*, *3b*, *3c*, *4c* longer (73–77) and thicker than other (32–41).

*Anogenital region*. All anogenital setae setiform, barbed. Six pairs of genital (*g*_1_, 20–24; *g*_2_–*g*_6_, 12) and two pairs of anal (*an*_1_, *an*_2_, 20–24) setae short; genital setae inserted in one longitudinal row. Eight pairs of aggenito-adanal setae present (from these: two pairs of aggenital setae, *ag*; three pairs of adanal setae, *ad*_1_–*ad*_3_; three pairs of additional, neotrichial setae, *n*). Two pairs of lateral neotrichial setae longer (53–61) than other (32–41). Lyrifissures *iad* located in inverse apoanal position, distanced from the anal plates.

*Legs*. Claw of each leg smooth. Morphology of leg segments, setae and solenidia typical for *Ctenobelba* (Ermilov et al. 2012b). Formulae of leg setation and solenidia: I (1–5–3–4–20) [1–2–2], II (1–5–3–4–15) [1–1–2], III (2–3–2–3–15) [1–1–0], IV (1–2–3–3–12) [0–1–0]; homology of setae and solenidia indicated in Table 1. Setae setiform, barbed (except smooth *s* on tarsi I and *p*). Famulus (*e*) short, thin. Solenidia slightly thickened, blunt-ended.

**Figures 1–2.**
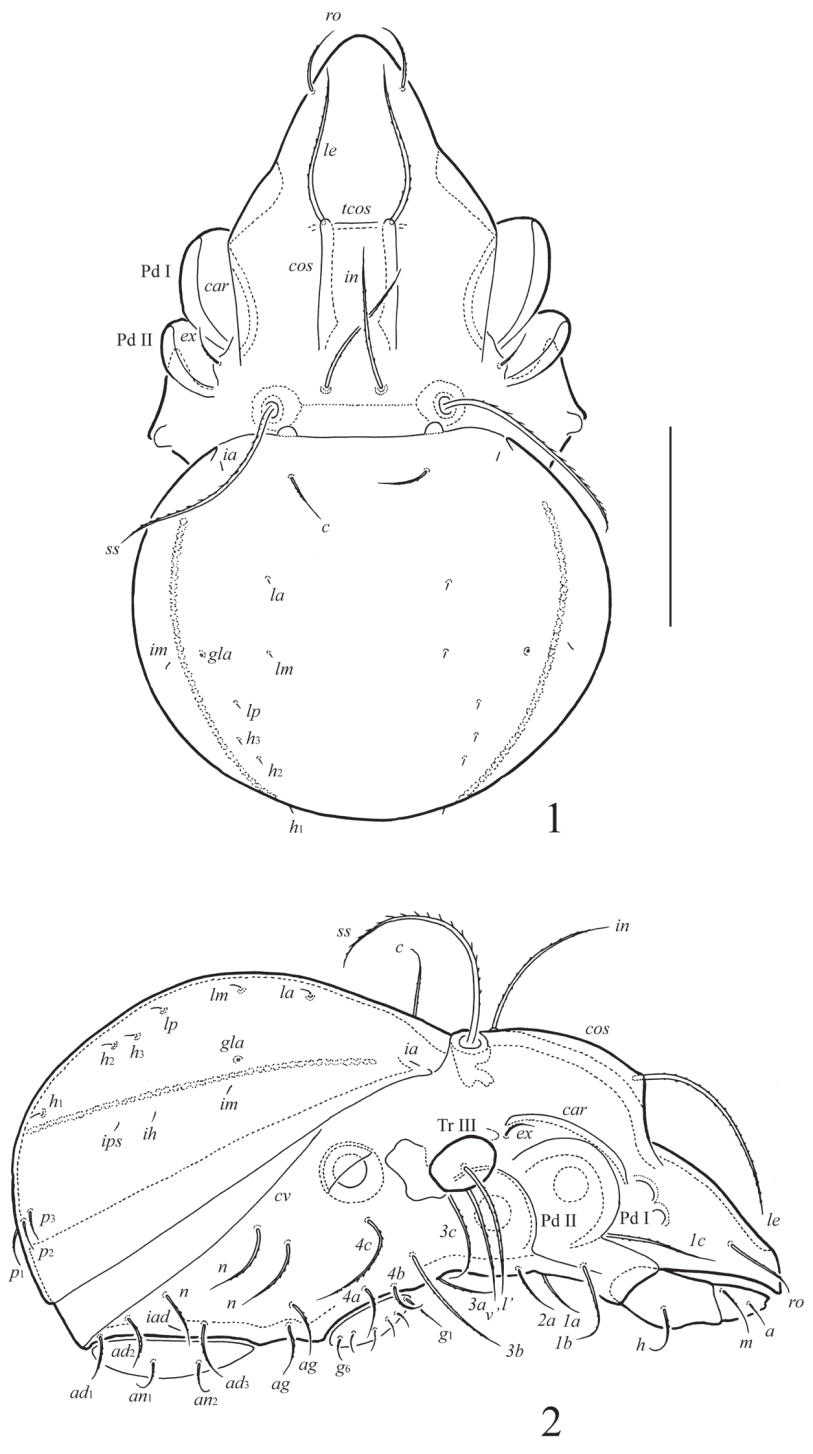
*Ctenobelba (Berndamerus) bugiamapensis* sp. n.: **1** dorsal view (legs not shown) **2** lateral view (legs except trochanter III not shown). Scale bar 100 μm.

**Figures 3–4.**
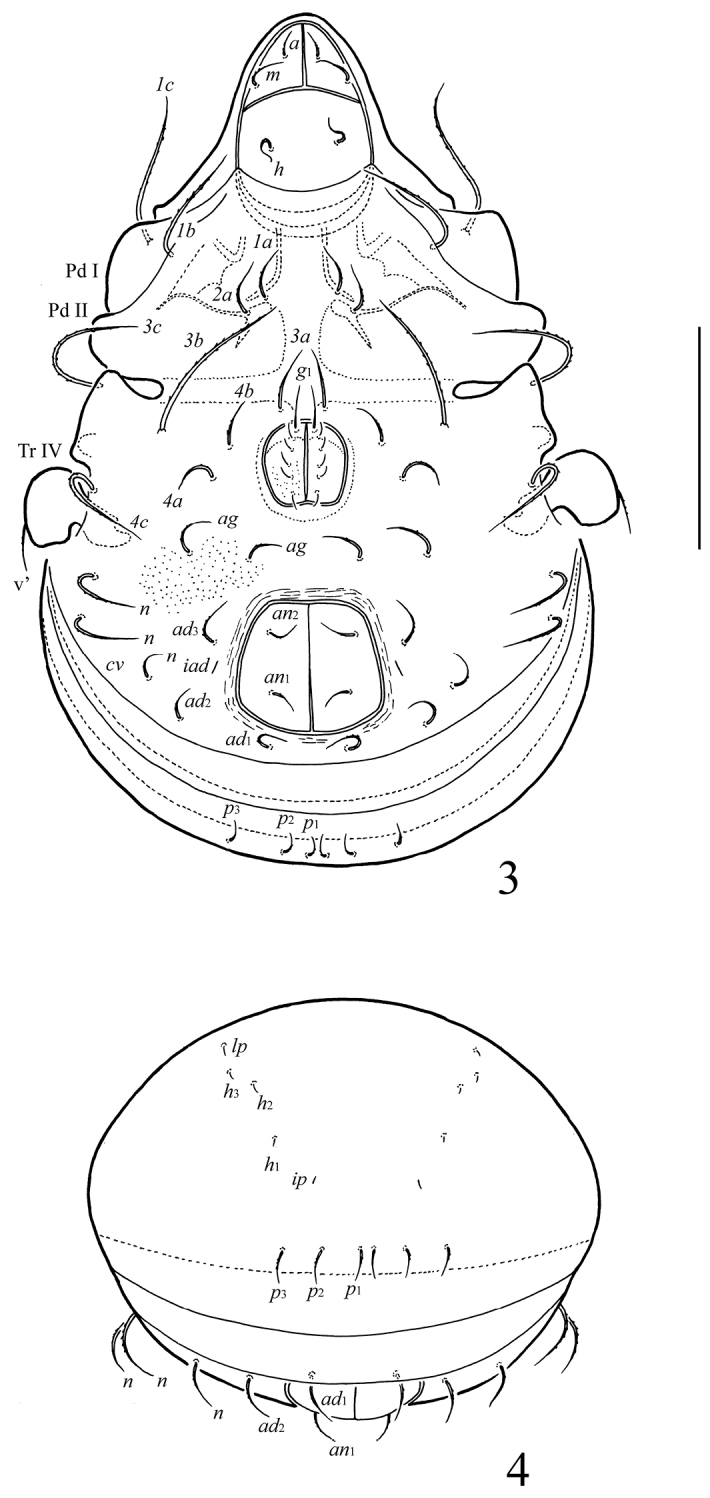
*Ctenobelba (Berndamerus) bugiamapensis* sp. n.: **3** ventral view (legs except trochanter IV not shown) **4** posterior view. Scale bar 100 μm.

**Figures 5–9.**
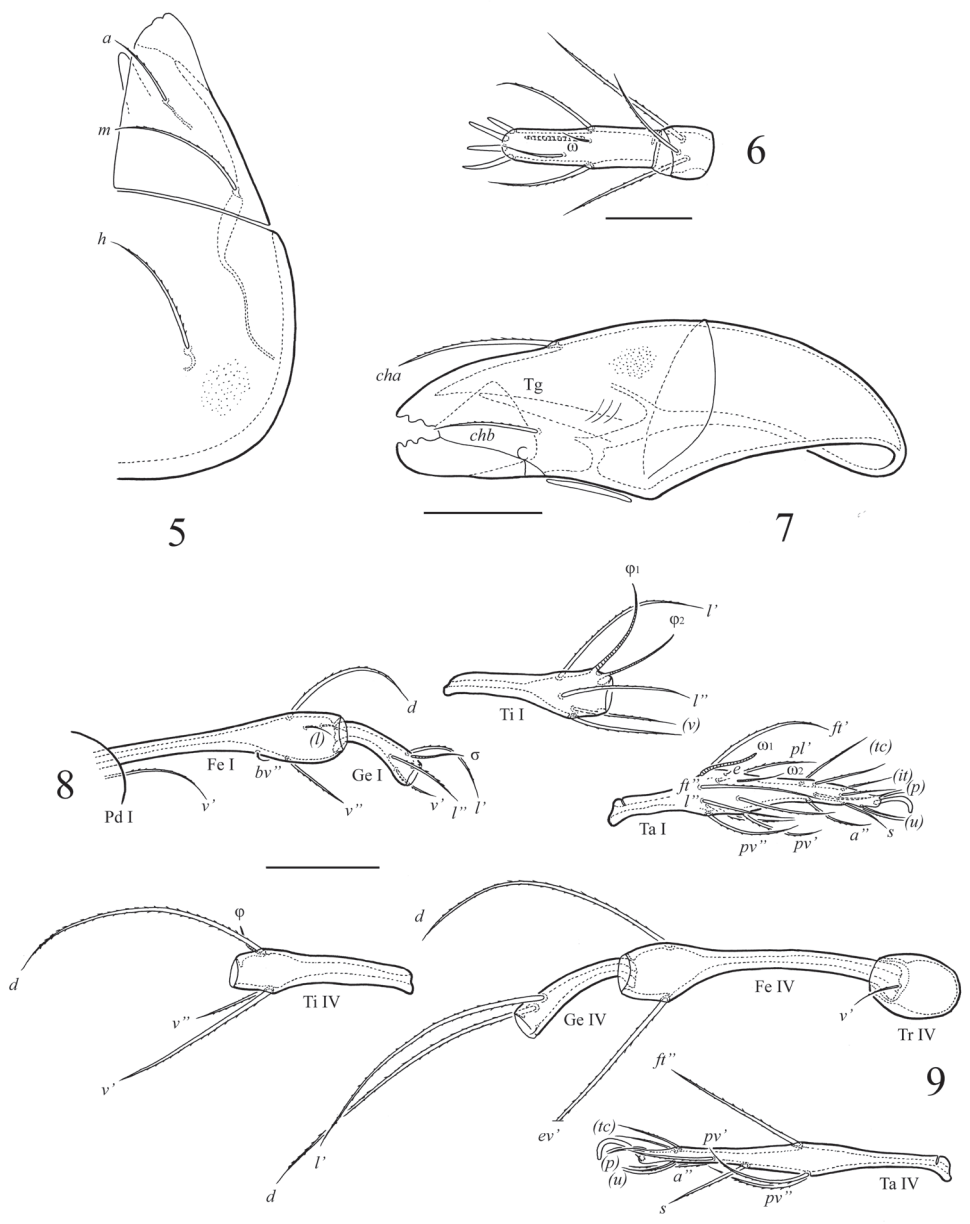
*Ctenobelba (Berndamerus) bugiamapensis* sp. n., adult: **5** subcapitulum, left half, ventral view **6** tibia and tarsus of palp **7** chelicera **8** leg I (trochanter and basal part of femur not shown), right, antiaxial view **9** leg IV, right, antiaxial view. Scale bar 20 μm (5, 7); 10 μm (6); 50 μm (8–9).

**Table 1.** Leg setation and solenidia of adult and tritonymph *Ctenobelba (Berndamerus) bugiamapensis* sp. n.

Leg	Trochanter	Femur	Genus	Tibia	Tarsus
I	*v*’	*d*, (*l*), *bv*’’, *v*’’	(*l*), *v*’, σ	(*l*), (*v*), φ_1_, φ_2_	(*ft*), (*tc*), (*it*), (*p*), (*u*), (*a*), *s*, (*pv*), *v*’, (*pl*), *l*’’, *e*, ω_1_, ω_2_
II	*v*’	*d*, (*l*), *bv*’’, *v*’’	(*l*), *v*’, σ	(*l*), (*v*), φ	(*ft*), (*tc*), (*it*), (*p*), (*u*), (*a*), *s*, (*pv*), ω_1_, ω_2_
III	*l*’, *v*’	*d*, *l*’, *ev*’	*l*’, *v*’, σ	*l*’, (*v*), φ	(*ft*), (*tc*), (*it*), (*p*), (*u*), (*a*), *s*, (*pv*)
IV	*v*’	*d*, *ev*’	*d*, *l*’, *v*’	*l*’, (*v*), φ	*ft*’’, (*tc*), (*p*), (*u*), (*a*), *s*, (*pv*)

Roman letters refer to normal setae (*e* to famulus), Greek letters to solenidia. Single prime (’) marks setae on anterior and double prime (’’) setae on posterior side of the given leg segment. Parentheses refer to a pair of setae.

##### Type deposition.

The holotype (ethanol) is deposited in the collection of the Zoological Institute of the Russian Academy of Sciences, St. Petersburg, Russia; two paratypes (ethanol) are deposited in the collection of the Siberian Zoological Museum, Novosibirsk, Russia; three paratypes (ethanol) are deposited in the collection of the Tyumen State University Museum of Zoology, Tyumen, Russia.

##### Etymology.

The specific name “*bugiamapensis*” refers to the Vietnamese park of origin, Bu Gia Map National Park.

##### Comparison.

The new species is most similar to *Ctenobelba (Berndamerus) eremuloides* (Berlese, 1910) comb. n. in having the lamellar setae longer than rostral and interlamellar setae and the localization of notogastral setae, however, it differs from the latter by the smooth rostrum (versus with teeth), heterotrichy of notogastral setae (versus heterotrichy absent), aggenito-adanal region with eight pairs of setae (versus with six), and adanal lyrifissures located in inverse apoanal position (versus apoanal position).

### New diagnosis of the family Ctenobelbidae

Prodorsum with long, parallel one pair of costulae. Rostrum rounded or dentate. Lamellar setae inserted on the costular ends. Sensilli bifurcate or setiform, with cilia (three to 25) or long branches unilaterally. Tutoria absent. Cerotegument presented by granules or reticulate ornamentation, rarely absent. Anterior margin of notogaster with one pair of tubercles. Ten pairs of notogastral setae present; they of diverse shape (thickened or leaf-like or with long, bent thin tip) or simple. Pedotecta I and II well developed. Epimeral formula: 3–1–3–3 (rarely: 3–1–3–4 or 3–1–4–4); epimeral setae *1b* often longest, directed forward. Six pairs of genital, two to five aggenital (rarely more than five), two pair of anal and three pairs of adanal setae present (rarely more aggenital and adanal setae). Lyrifissures *iad* located posteriad to adanal setae *ad*_3_. Solenidia of tibiae and genua not coupled with dorsal seta. Legs monodactylous.

Type genus: *Ctenobelba* Balogh, 1943. The genus having morphological features of the family.

### Key to known subgenera of *Ctenobelba*

**Table:** 

1	Aggenito-adanal region with strong neotrichy (more than 20 pairs of setae present)	subgenus *Ctenobelba (Aokibelba)* Subías & Shtanchaeva, 2013
–	Aggenito-adanal region with less than nine pairs of setae	2
2	Sensilli bifurcate	subgenus *Ctenobelba (Bifurcobelba)* Subías & Shtanchaeva, 2010
–	Sensilli setiform, with long branches or cilia unilaterally	3
3	Body surface reticulate	subgenus *Ctenobelba (Caucasiobelba)* Subías & Shtanchaeva, 2010
–	Body surface not reticulate	4
4	Body surface without cerotegument; lamellar setae inserted at a distance from rostral and interlamellar setae on similar length	subgenus *Ctenobelba (Berndamerus)* Mahunka, 1977
–	Body surface with cerotegument; lamellar setae inserted close to rostral setae and at a distance from interlamellar setae	subgenus *Ctenobelba (Ctenobelba)* Balogh, 1943

### Key to known species of *Ctenobelba (Berndamerus)*

**Table:** 

1	Heterotrichy of notogastral setae present: *c* and *p*_1_–*p*_3_ of medium size, other setae minute; aggenito-adanal region with eight pairs of setae (including two pairs of aggenital setae), adanal neotrichy present; adanal lyrifissures located in inverse apoanal position; body size: 315–365 × 199–232	*Ctenobelba (Berndamerus) bugiamapensis* sp. n. (description see in this paper; distribution: southern Vietnam)
–	Heterotrichy of notogastral setae absent: all setae of medium size, similar in length; aggenito-adanal region with six to seven pairs of setae (including three to four pairs of aggenital setae), adanal neotrichy absent; adanal lyrifissures located in paraanal or apoanal position	2
2	Rostrum with several teeth; lamellar setae longer than rostral and interlamellar setae; adanal lyrifissures located in apoanal position; body size: 450 × 220	*Ctenobelba (Berndamerus) eremuloides* (Berlese, 1910), comb. n. (description see in Berlese (1910b), Mahunka and Mahunka-Papp (1995); distribution: southern Europe)
–	Rostrum rounded; lamellar setae shorter than rostral and interlamellar setae; adanal lyrifissures located in paraanal position	3
3	Notogastral setae *la* inserted posteriorly to *c*, *h*_3_ inserted antero-laterally to *h*_2_, *h*_2_ distanced form *lm*; body size: 580 × 300	*Ctenobelba (Berndamerus) bicostata* (Berlese, 1910), comb. n. (description see in Berlese (1910a), Mahunka and Mahunka-Papp (1995); distribution: Mediterranean)
–	Notogastral setae *la* inserted latero-posteriorly to *c*, *h*_3_ inserted anteriorly to *h*_2_, *h*_2_ and *lm* located close to each other; body size: 648–664 × 328–344	*Ctenobelba (Berndamerus) hellenica* (Mahunka, 1977), comb. n. (description see in Mahunka (1977); distribution: Greece)

## Supplementary Material

XML Treatment for Ctenobelba (Berndamerus) bugiamapensis
